# Automating the triage of rheumatology outpatient referrals: a comparative evaluation of 23 large language models under simple and advanced prompting

**DOI:** 10.1007/s00296-026-06317-8

**Published:** 2026-10-09

**Authors:** Lynden Roberts

**Affiliations:** 1 Faculty of Medicine, Nursing, and Health Sciences, Monash University, Clayton, Australia; 2 Digital & Information Division, Monash Health, 246 Clayton Rd, Melbourne, VIC 3168 Australia; 3 Department of Rheumatology, Monash Health, Melbourne, VIC Australia

**Keywords:** Rheumatology, Referral and consultation, Triage, Artificial intelligence, Natural language processing, Health services research

## Abstract

Triage of rheumatology outpatient referrals is a high-volume administrative task that consumes senior specialist time without advancing patient care. The human triage system is only moderately accurate and reproducible. We assessed whether contemporary large language models (LLMs) are able to perform well enough to support automating this task in practice. In addition, the effects of different prompting techniques on triage accuracy and cost was assessed to help identify the optimal approach. Twenty referral scenarios spanning the urgency spectrum were created by a certified Australian rheumatologist. Four rheumatologists triaged all cases independently and blinded to produce a consensus reference standard; inter-rater reliability was quantified. Twenty-three LLMs each triaged every referral into one of five urgency categories, three times (1380 outputs per referral). The experiment was run with a simple prompt and repeated with an advanced prompt supplying explicit triage expectations and worked examples. In line with published literature, agreement among the four rheumatologists was moderate (Fleiss kappa 0.60; mean pairwise linear-weighted kappa 0.79), with all four assigning categories within one level of each other in 90% of cases. All 2760 model calls returned an interpretable result. Under the simple prompt, performance separated into distinct tiers, larger models were more accurate (Spearman rho = 0.42; *P*=.047) and accuracy tracked cost. Advanced prompting reduced between-model variance in accuracy 5.3-fold (0.014 to 0.003; Levene *P*=.01), abolished the size-accuracy association (rho=-0.05; *P*=.83) and removed the accuracy-cost relationship. The best models matched expert consensus on 18 of 20 cases, comparable to the reproducibility observed among the rheumatologists themselves. Under-triage errors persisted with some LLMs. Contemporary LLMs categorised rheumatology referral urgency with an accuracy and reproducibility comparable to that reported for human triage systems, although human triage was not tested in this study. Advanced prompting appeared to substitute for the reasoning capability of larger models, suggesting that adequate performance on this task may not require the most expensive models. These findings indicate that automation of this administrative task is technically feasible. Candidate models that warrant prospective evaluation are identified.

## Introduction

Safe access to rheumatology outpatient care depends on triage: the sorting of incoming referrals by clinical urgency so that finite specialist capacity is directed first to those at greatest risk of harm from delay. In most services this is done by a consultant rheumatologist or senior registrar reading free-text referral letters. It is a high volume, cognitively demanding, and it a time-consuming task using clinician resources that does not itself advance a patient’s care or generate any therapeutic outcome [1, 2]. Every hour spent triaging is an hour lost to direct clinical work. As referral volumes rise against a rheumatology workforce that is widely projected to fall short of demand [3], this opportunity cost has become a pressing operational problem for the specialty rather than a peripheral administrative irritation.

The rheumatology casemix exemplifies the risks and costs of referral triage systems. The spectrum reaching a clinic ranges from organ- or sight-threatening emergencies such as giant cell arteritis, systemic vasculitis and lupus with organ involvement, through to degenerative and non-inflammatory conditions that can safely wait months [4]. Getting urgency wrong carries real consequences: delays in the assessment of early inflammatory arthritis are associated with worse long-term joint and functional outcomes [5].

Manual triage is also an imperfect standard against which to judge an automated alternative. In one rheumatology series the urgency grade assigned from the referral changed after specialist consultation in almost half of cases, and roughly one in six truly urgent patients were under-triaged at the referral stage, creating avoidable clinical risk [6]. A structured rheumatology referral tool detected urgent presentations with a sensitivity and specificity of 80% and 79% respectively, leading to problematically high rates of misclassification [7]. Reproducibility is similarly moderate: independent clinicians prioritising identical referrals typically agree only moderately (weighted kappa about 0.6), disagree on roughly a third of cases, and do not improve appreciably with training [8, 9], while specialists vary widely when triaging identical referrals across settings [10]. The pertinent question for an automated aid is therefore not whether it is perfect, but whether it matches or exceeds a human standard that is itself only moderately accurate and reproducible - particularly given automation advantages of speed, turnaround, consistency and cost.

Published approaches to automated triage have relied on conventional natural language processing and machine learning applied to referral letters, delivering modest, narrowly scoped gains insufficient for rollout at scale [4, 11]. Large language models (LLMs) appear better suited: they encode broad clinical knowledge, pass licensing examinations, and can support diagnostic and triage decisions across specialties [12–14]. Early referral-triage evaluations in other specialties report useful but imperfect accuracy [15], and within rheumatology LLMs have approached specialist performance on diagnostic tasks [16]. A recurring theme is that how a model is prompted - chain-of-thought reasoning, worked examples, guided reflection - can matter as much as which model is used [17–19]. No published study has yet interrogated how prompt design interacts with model capability and cost across a broad contemporary panel on specialist urgency triage.

This gap has direct economic consequences for deployment at scale. If triage accuracy tracks model size and price, achieving affordability may require acceptance of poorer performance; if a well-specified prompt can raise smaller, cheaper models to competitive accuracy, the economics of a deployable triage assistant may prove viable. We therefore benchmarked 23 contemporary LLMs on the urgency triage of typical rheumatology outpatient referrals against a blinded four-rheumatologist consensus, comparing their performance, its relationship to serving cost, and the effect of two prompting strategies.

## Methods

### Study design and setting

A comparative evaluation study at a specialist rheumatology clinic within an Australian academic healthcare network was conducted. The task under evaluation was the assignment of an urgency triage category to a general practitioner (GP) referral, mirroring the administrative decision made daily in the service. Every model triaged every case, each case was attempted three times, and the entire experiment was repeated under two prompt conditions. Thus, 23 models x 20 cases x 3 attempts = 1380 model outputs per prompt condition, and 2760 outputs in total.

### Case selection and development

Twenty referrals representative of the range of presentations typically received by the clinic from Australian GPs were constructed by a certified rheumatologist, who has personally triaged many thousands of such referrals. Each scenario was created to be consistent with real referral letters in content, clinical detail and level of completeness. Though a systematic process was not applied, the set was intentionally designed to span the full urgency spectrum and the usual conditions referred: from organ- or life-threatening inflammatory and autoimmune disease requiring near-immediate review, through active but stable inflammatory conditions, to degenerative and non-inflammatory presentations appropriate for routine scheduling.

### Triage categories and definitions

Each referral was assigned to one of five mutually exclusive urgency categories, defined by the maximum safe waiting time: Emergency (seen within 1 day), Hyper-Urgent (within 7 days), Urgent (within 1 month), Semi-Urgent (within 3 months) and Routine (within 12 months). The governing principle, applied by both human experts and models, was to select the category that achieved the safest longest wait, without risking death or permanent damage.

### Gold-standard consensus

The reference standard for each case was established by four rheumatologists who each triaged all 20 referrals independently, blinded to one another’s decisions and to model outputs. A single consensus urgency category per case was then agreed and used as the gold standard against which all model outputs were scored. For 8 of the 20 cases the expert reference concluded that either of two adjacent categories represented correct triage; for these cases a model was scored as correct if it selected either category.

Inter-rater reliability among the four rheumatologists was quantified before consensus was reached, using their independent ratings. Where a rheumatologist nominated two adjacent categories, the more urgent was taken as the primary rating for the reliability analysis, with the less urgent used in a sensitivity analysis. Agreement was summarised as Fleiss kappa across all four raters, mean pairwise linear-weighted Cohen kappa, exact pairwise agreement, and the proportion of cases in which all four raters agreed exactly or fell within one adjacent category.

### Language models and serving platform

Twenty-three contemporary LLMs were evaluated, and the identical model set was used in both prompt conditions. Models were accessed through a single application programming interface aggregator (OpenRouter), which provides unified access to models from multiple providers. The evaluated models were: anthropic/claude-fable-5, anthropic/claude-haiku-4.5, anthropic/claude-opus-4.8, anthropic/claude-sonnet-5, deepseek/deepseek-v4-pro, google/gemini-2.5-flash, google/gemini-2.5-pro, google/gemini-3.1-pro-preview, google/gemini-3.5-flash, meta-llama/llama-4-maverick, minimax/minimax-m3, mistralai/mistral-medium-3-5, moonshotai/kimi-k2.6, nvidia/nemotron-3-ultra-550b-a55b, openai/gpt-5.4-nano, openai/gpt-5.5, openai/o1, openai/o3, openai/o4-mini-high, qwen/qwen3.6-flash, qwen/qwen3.7-max, x-ai/grok-4.3 and z-ai/glm-5.2. Hence, the panel spanned a wide range of model families, sizes, release generations and serving prices, including both large frontier reasoning models and smaller, lower-cost models.

Experiments were run via a purpose-built bulk-testing platform (internally developed web application) designed to test prompts, cases and models at scale. The platform issued each model-case-attempt request, captured the model’s chosen category and free-text rationale, and logged latency, prompt and completion token counts, total tokens and serving cost for every attempt. Serving cost is reported as the price charged by the aggregator for model inference, in US dollars (USD).

### Prompt conditions

Two prompts were evaluated (full text in Table 1). The simple prompt provided minimal task instructions. The advanced prompt added explicit direction reflecting Australian triage expectations, instructed chain-of-thought reasoning, directed the model to focus on biological disease by disregarding pain severity and psychosocial impact, and supplied worked examples of conditions belonging to each category.

**Table 1:** Full text of the two prompt conditions evaluated

Simple prompt	“You are an expert rheumatologist who receives an outpatient referral from a GP and outputs the correct triage category for the referral. Triage categories are: ‘Emergency’ <1 day, ‘Hyper-Urgent’ <7 days, ‘Urgent’ <1 month, ‘Semi-Urgent’ <3 months, ‘Routine’ <12 months. Consider each category for the referral one-by-one, and then choose the best fit for safely waiting as long as possible. Output the correct triage category.”
Advanced prompt	“You are an expert rheumatologist who receives an outpatient referral from a GP and outputs the correct triage category for the referral. Your job is to make patients wait as long as possible without risking death or damage to a vital organ. You use chain of thought reasoning to analyse the problem step by step. You use a scratchpad to record your thinking. Be ruthless in completely ignoring pain severity, psychological or social impacts of the condition. Focus only on the rheumatological condition. Triage categories are: ‘Emergency’ 1 day, ‘Hyper-Urgent’ 7 days, ‘Urgent’ 1 month, ‘Semi-Urgent’ 3 months, ‘Routine’ 12 months. Consider each category for the referral one-by-one, and then choose the best fit for safely waiting as long as possible. As a guide, Emergency would include (SLE with organ involvement, GCA with threatened vision, vasculitis with organ involvement, septic arthritis); Urgent would include (rheumatoid arthritis with severe functional impairment); Semi-Urgent would include (persistent disabling active gout, any poorly controlled inflammatory arthritis, any active autoimmune connective tissue disease without organ involvement); Routine would include (all degenerative conditions, osteoarthritis, fibromyalgia, well controlled autoimmune or inflammatory conditions, positive autoimmune serology without clinical symptoms). Output the correct triage category and brief explanation of why they can’t wait longer.”

GCA giant cell arteritis, GP general practitioner, SLE systemic lupus erythematosus

To assess whether the advanced prompt could have leaked answers, the worked examples were reviewed against the test cases. The worked examples consisted only of short condition labels (for example ‘giant cell arteritis with threatened vision’, ‘osteoarthritis’); no case vignette or any part of one appeared in either prompt, and the cases were written before the prompts. Seventeen of 20 cases shared at least one broad diagnostic concept with a worked example, which is unavoidable when examples are intended to define category boundaries. In five cases the correct consensus grade did not match the urgency implied by the most closely aligned worked example. For example, 2 acute monoarthritis presentations that the GP were suggestive of crystal arthritis were graded Emergency because septic arthritis still had to be excluded; sicca with positive serology and widespread pain with a positive antinuclear antibody were graded Routine; and lupus-associated serology without organ involvement was graded Semi-Urgent. The advanced prompt therefore cannot have leaked the correct grades through the worked examples: matching a case to an example because they share a diagnosis or a laboratory term would have produced the wrong urgency in these five cases, not the right one.

### Repeat runs

To test output stability, each model triaged each case three times, separately and independent of prior result knowledge, within each prompt condition. Repeated sampling allowed each model decision to be summarised as a confidence profile rather than a single point estimate, distinguishing decisions reached unanimously across attempts from those reached less consistently.

### Outcomes and scoring

The primary outcome was triage accuracy relative to expert consensus. For each model and case, the three attempts were collapsed into a repeat-run confidence profile with five mutually exclusive outcomes: high-confidence correct (matched consensus on all three attempts, 3/3); lower-confidence correct (matched on 2 of 3); miss by over-triage (more urgent category than the expert); miss by under-triage (less urgent category than the expert); and unclassified or error. A case was counted as correct when the model’s category matched consensus on the majority of runs, and per-model accuracy was the proportion of correct cases. The distinction between over- and under-triage was made because the clinical risk is asymmetric: over-triage is safer, whereas under-triage exposes a patient to potentially harmful delay.

Models were ranked by total correct (3/3 + 2/3), then by high-confidence correct, then by fewer over-triage misses, fewer under-triage misses, and fewer unclassified or error outcomes; remaining ties were broken by lower total run cost.

### Statistical analysis

**Fig. 1:**
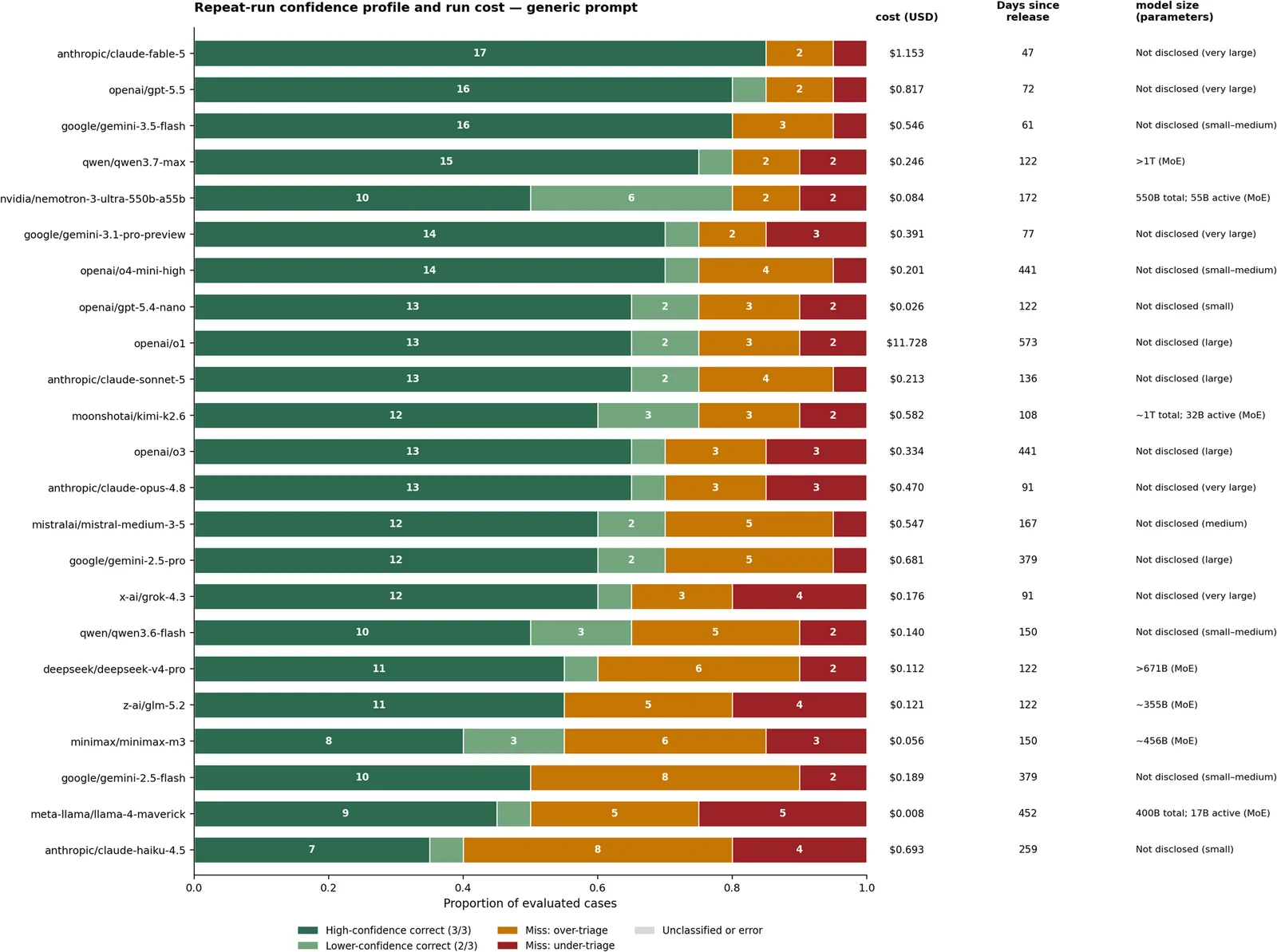
Triage performance of 23 large language models under the simple prompt. Each row shows one model. Stacked bars show the repeat-run confidence profile across three attempts per referral: high-confidence correct (matched expert consensus on 3/3 attempts), lower-confidence correct (2/3), miss by over-triage (more urgent category than consensus), miss by under-triage (less urgent category than consensus), and unclassified or error. Right-hand columns show total serving cost in US dollars (USD), days since the model’s public release calculated to 1 July 2026, and model size in parameters (best available estimate; inequality signs denote undisclosed commercial models). Models are ranked by total correct

**Fig. 2:**
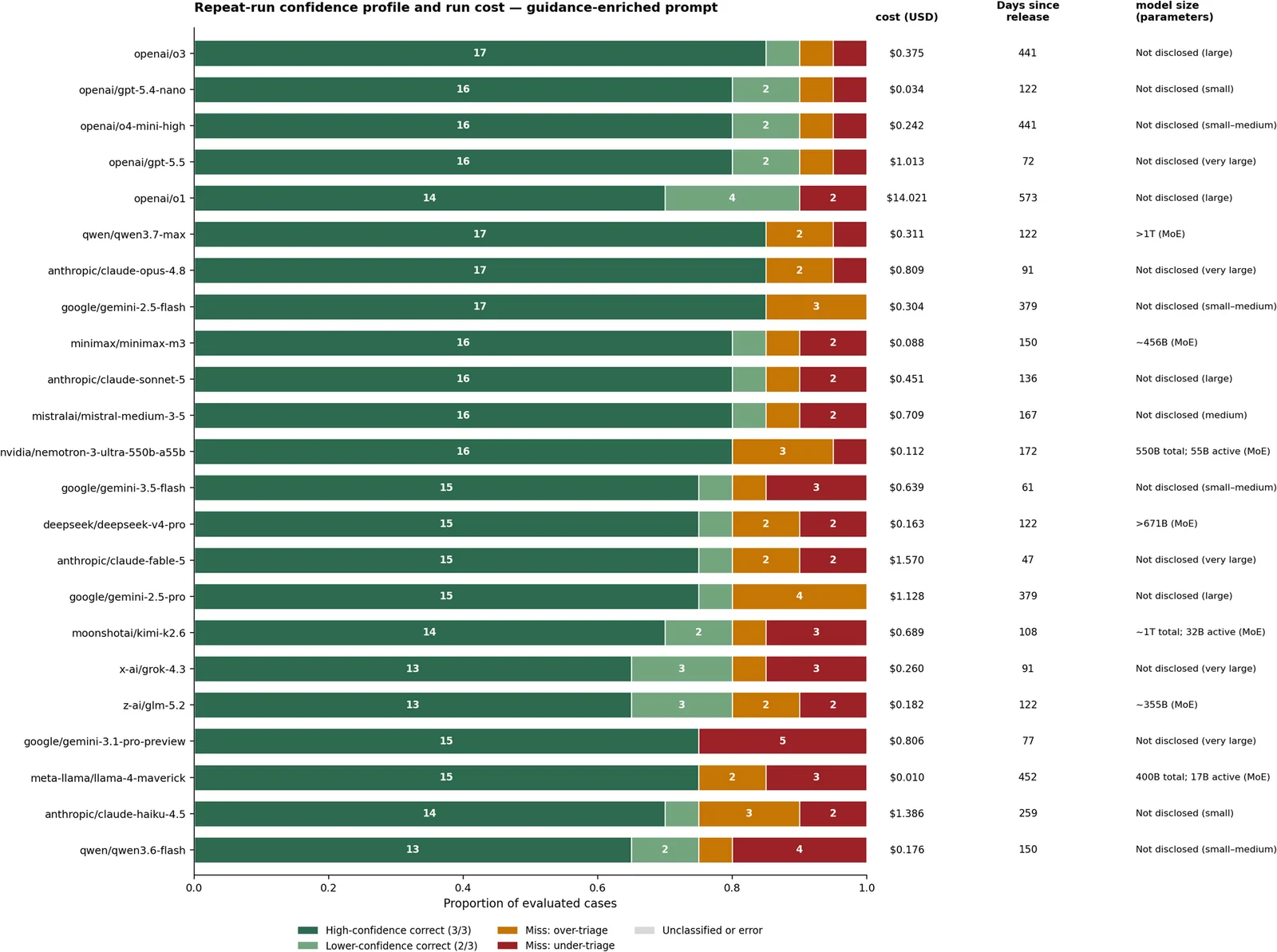
Triage performance of the same 23 large language models under the advanced prompt. Categories, right-hand columns and ranking are as in Fig. 1. Days since release are calculated to 1 July 2026 and cost is reported in US dollars (USD). Between-model variance in accuracy is markedly compressed relative to Fig. 1

**Fig. 3:**
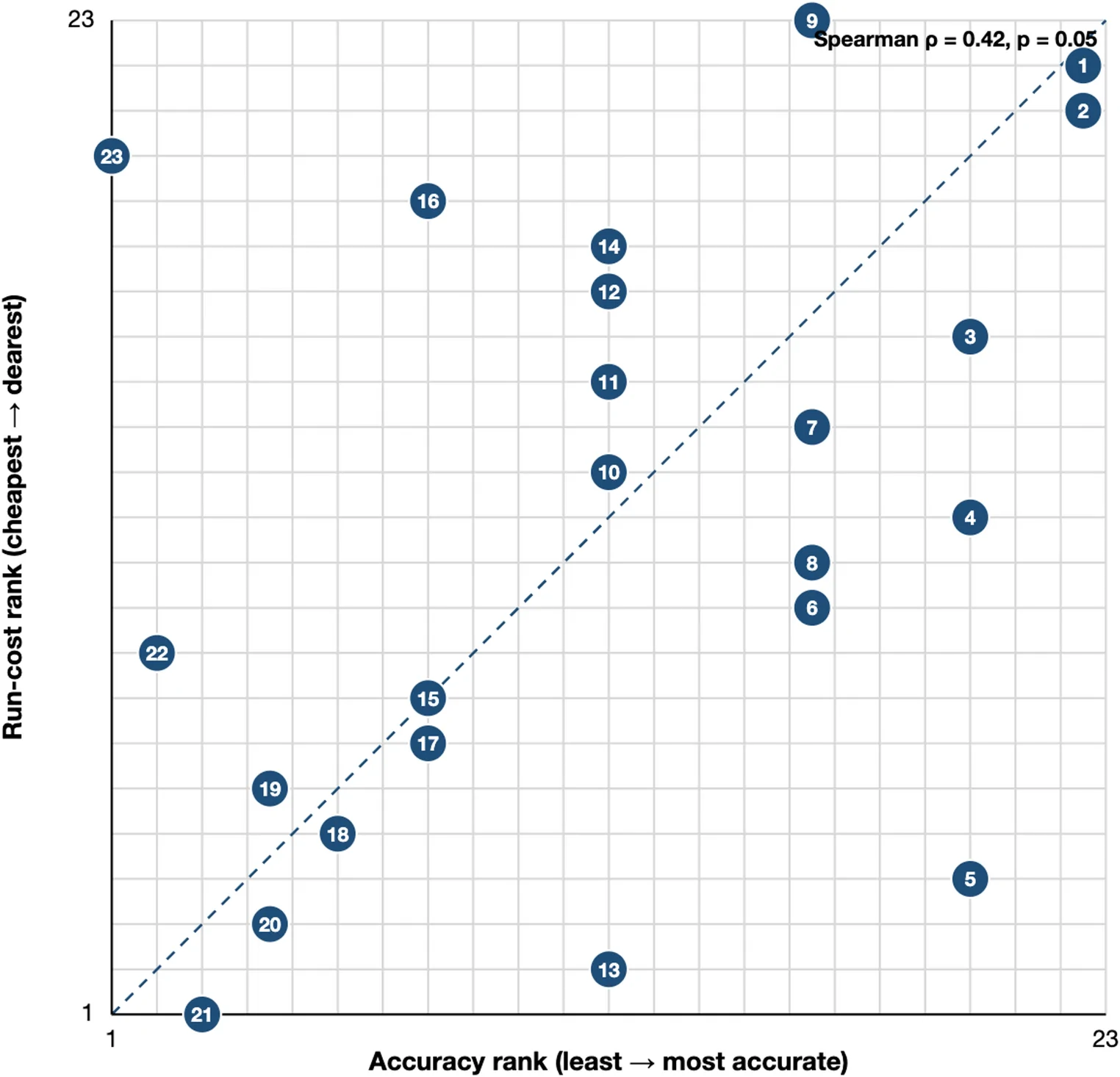
Association between per-model triage accuracy and per-model serving cost under the simple prompt. Each point represents one model. Accuracy increased with cost (Spearman rho=0.42; P=.05)

**Fig. 4:**
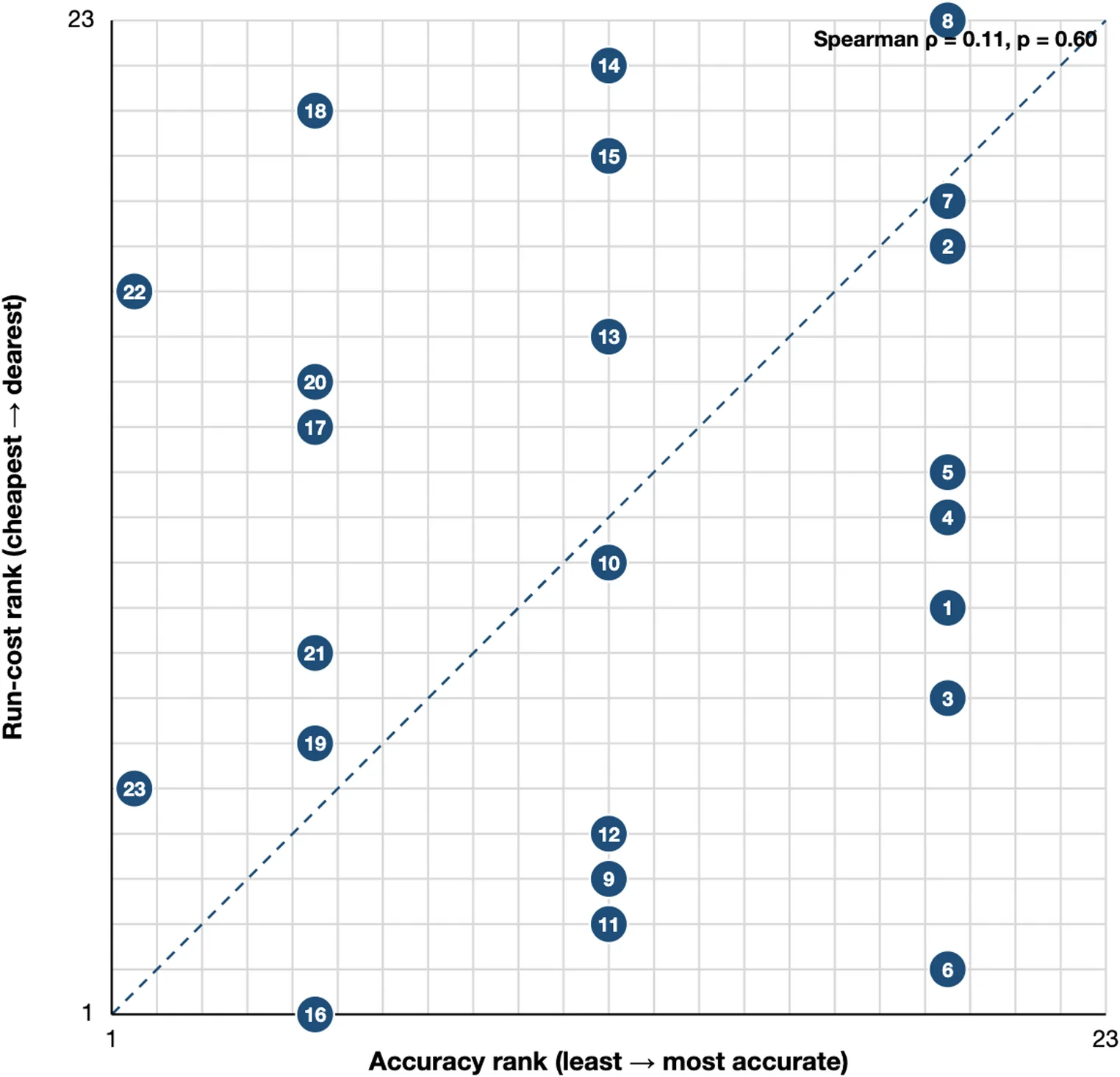
Association between per-model triage accuracy and per-model serving cost under the advanced prompt. Each point represents one model. No systematic accuracy advantage was seen for higher-cost models (Spearman rho=0.11; P=.60)

Results were summarised descriptively and visually. For each prompt condition we examined the distribution of confidence-profile outcomes across the 23 models (Figs. 1 and 2) and the relationship between per-model accuracy and per-model serving cost (Figs. 3 and 4). Associations between model size, cost and accuracy were assessed with Spearman rank correlation, though these correlations should be considered exploratory. Confidence intervals for the correlations were estimated by bootstrap resampling of the 23 models (percentile method). Equality of between-model variance in accuracy across the two prompt conditions was tested with the Levene test, with the Bartlett test as a sensitivity analysis. Agreement among the four rheumatologists was assessed with Fleiss kappa and linear-weighted Cohen kappa. Two-sided P values below 0.05 were considered statistically significant.

### Ethical considerations

The study was conducted under an ethics waiver governed by the institutional Human Research Ethics Committee and followed the principles of the Declaration of Helsinki. No patient-identifiable information was provided to any external model provider.

## Results

### Overview

All 1380 model-case-attempts in each prompt condition returned a triage category. Performance varied widely across the LLMs, and both the level of performance and its relationship to model cost differed markedly between the two prompt conditions. Agreement among the four rheumatologists triaging the same 20 referrals was moderate (Fleiss kappa 0.596). Exact pairwise agreement was 68.3%, rising to 98.3% when agreement to within one adjacent category was accepted. All four rheumatologists chose an identical category in 50% of cases and fell within one adjacent category in 90%. Nine of 20 cases prompted at least one rheumatologist to nominate two adjacent categories. The reference standard against which the models were judged therefore carries its own uncertainty.

### Model capability spanned a wide range under the simple prompt

Under the simple prompt, the LLMs separated into clearly distinct tiers of performance (Fig. 1). Larger and more recent models achieved the highest proportions of correct cases, with a substantial share of their correct answers reached unanimously across all three attempts. Smaller and older models performed considerably less well, with fewer total correct cases, a smaller unanimous fraction and a greater burden of misclassification. The spread between the strongest and weakest models was large, indicating that, when given simple instruction, the ability to translate a free-text referral into a safe urgency category was variable across contemporary LLMs. Across the LLMs, larger models achieved higher accuracy under this prompt (Spearman rho = 0.42; 95% CI 0.01 to 0.72; *P*=.047).

### Advanced prompting levelled performance across the panel

Repeating the experiment with the advanced prompt narrowed the gap between models substantially (Fig. 2). Between-model variance in accuracy fell from 0.014 to 0.003, a 5.3-fold reduction (Levene test *P*=.01; Bartlett test *P*<.001). The smaller and older models that had lagged under the simple prompt improved considerably once the prompt supplied explicit triage expectations and worked category examples, moving closer to the leading models in total correct cases and in the proportion of high-confidence correct decisions. The strongest models remained strong, but their advantage was negated. The positive association between model size and accuracy seen under the simple prompt disappeared (Spearman rho=-0.05; *P*=.83).

### Accuracy was associated with cost under the simple prompt

Plotting per-model accuracy against per-model serving cost under the simple prompt revealed a positive association (Spearman rho = 0.42; 95% CI 0.02 to 0.71; *P*=.047). More expensive models tended to achieve higher accuracy (Fig. 3). With minimal prompting instruction, therefore, paying more for a larger, more capable model bought better triage performance, and cost served as a partial proxy for accuracy on this task.

### The accuracy-cost association disappeared under advanced prompting

The same relationship was absent under the advanced prompt (Fig. 4). With explicit expectations and worked examples in the prompt, higher-cost models no longer showed a systematic accuracy advantage over lower-cost models (Spearman rho = 0.11; 95% CI -0.35 to 0.53; *P*=.60). This dissociation is a key finding: advanced prompting techniques not only raised the performance of weaker models but severed the link between how much a model cost and how accurately it triaged.

### Errors were distributed between over- and under-triage

Across both conditions, residual misclassification was not confined to the safer direction. The confidence profiles (Figs. 1 and 2) show that misses comprised both over-triage, in which a model assigned a more urgent category than the expert consensus, and under-triage, in which it assigned a less urgent category. Because under-triage is the direction that exposes a patient to potentially harmful delay, the persistence of under-triage errors - even among otherwise high-performing models and even under the advanced prompt - is the most safety-relevant feature of the results and directly shapes the requirements for any deployed system.

## Discussion

### Principal findings

We benchmarked 23 contemporary LLMs on the urgency triage of rheumatology outpatient referrals against a blinded four-rheumatologist consensus under two prompts. Four findings stand out. First, performance varied widely: contemporary LLMs are not interchangeable on specialist triage. Second, an advanced prompt with explicit expectations and worked examples produced a levelling effect, improving weaker, smaller and older models and compressing the performance gap 5.3-fold. Third, accuracy tracked serving cost under the simple prompt but not under the advanced prompt. Fourth, the leading models matched the consensus on most cases - unanimously across repeated runs for many - placing them within or above the range reported for human referral triage [6–10].

An interpretation is that under minimal instruction the model must supply, from its own capacity, both the clinical reasoning and the implicit triage policy needed to convert a referral into a safe category. Larger, more recent, more expensive models hold more of that latent capacity, so they perform better and cost more. When the prompt supplies the missing policy and reasoning scaffold, it substitutes for the capacity smaller models lack: the weaker models catch up and cost ceases to predict accuracy. Demonstrating this dynamic on a genuinely complex clinical-reasoning task is novel and practically consequential.

### Comparison with prior work

Our results extend evidence that prompt design can be as influential as model choice in clinical LLM applications. Structured prompting aligned model recommendations with an expert cardiology Heart Team far better than zero-shot prompting [18], and task-tailored, few-shot prompts raise performance on complex clinical tasks [19–21]. Our contribution is to show, across an unusually broad and contemporary LLM panel on a single specialist task, that advanced prompting not only raises average performance but specifically reduces the between-model differences and abolishes the accuracy-cost gradient - with direct implications for how services procure these models.

The findings also align with prior referral-triage work. Earlier studies showed LLMs could triage referrals with some accuracy, though performance remained uneven across presentations [15, 22]; machine-learning pipelines applied to GP letters could prioritise high-risk patients but were less effective when referrals were complex or ambiguous [4, 11]. Broader benchmarking finds capable but imperfect performance with both error directions and a consistent call for human oversight [14, 23]. Within rheumatology specifically, LLMs have already approached specialist performance on diagnostic reasoning [16], although their adherence to guideline criteria has been shown to drift between sessions, underlining the need for the repeat-sampling stability we measured here [24]; the present study extends that finding from diagnosis to the operational decision that actually governs access to care.

### Why this matters to rheumatology services

Triage is, from the patient’s perspective, invisible work. It consumes senior rheumatologist time, generates no diagnosis, no treatment and no outcome, and yet must be done accurately because the cost of error falls on patients with time-critical inflammatory and autoimmune disease [1, 2, 5]. It is precisely the sort of task that should be automated first: high volume, rule-governed, repetitive, and non-therapeutic. The barrier has never been desirability but evidence that automation is safe enough. The present data speak to that barrier. The strongest models agreed with a four-rheumatologist consensus on most cases and did so reproducibly across repeated sampling, which places them at or above the band reported for human referral triage - a standard where urgency grades are frequently revised after assessment, a significant minority of urgent patients are initially under-triaged [6, 7], and independent clinicians disagree on around a third of cases with little benefit from training [8–10]. Unlike a clinician, a model can be resampled, audited, and does not tire, or suffer the cognitive biases that affect clinical judgement [16, 25]. Models carry their own risks of encoded bias, which must be audited rather than assumed absent [26].

The second finding changes the economics. Because a well-specified, example-based prompt lets smaller, cheaper models perform competitively, an affordable and scalable triage assistant no longer requires access to the most expensive frontier model. For a rheumatology service, this makes automation realistic in the near term, and returns senior clinician hours to direct patient care at a time when workforce shortfalls are projected to worsen [3]. Our factorial platform offers a template for the local evaluation of prompts, cases and models that responsible deployment requires, including post-deployment auditing.

However, there are caveats. Our reference standard is itself human judgement rooted in one service’s norms, so ‘human-level’ means agreement with expert rheumatologists, not demonstrated superiority; and because the four rheumatologists themselves agreed exactly on only half of the cases, some of the reduced model performance reflects case ambiguity rather than model error. The comparison with human triage is with published benchmarks rather than a human control in this study, and those benchmarks arise from different settings. Residual under-triage, which is common in studies of human triage, persisted even in the best condition. The configuration of deployed system therefore should be biased towards over-triage, with mandatory clinician review of the most urgent categories, and continuous audit of under-triage rates.

### Limitations

This study used 20 constructed referrals at a single academic centre. Although written to span across the urgency and clinical spectrum, the heterogeneity and ambiguity of real-world GP referrals cannot have been fully captured here. With 20 cases, a single case would change the measured accuracy by 5% points, so small between-model differences should not be over-interpreted and the reported correlations should be considered exploratory. The reference standard, from four blinded rheumatologists, embeds one service’s triage norms, which legitimately differ between countries and between services in the same jurisdiction. Model versions and prices reflect a single time point in a fast-moving field; all evaluations were run in July 2026 and cost was serving price rather than a health-economic evaluation. Because models do not disclose their training data, contamination by similar published vignettes cannot be entirely excluded. Finally, we ran no contemporaneous head-to-head human arm, so the comparison with human triage rests on external benchmarks from varied settings and taxonomies [6–10].

### Future directions

Priorities follow from these limitations: prospective evaluation on a larger consecutive sample of real de-identified referrals; external validation across services with differing triage norms; systematic optimisation of the advanced prompt, including the number and selection of worked examples and self-consistency across repeated sampling [18, 27]; formal characterisation of under-triage risk and the human-oversight workflow needed to contain it; and prospective assessment of clinician time saved and effects on waiting times. Given the pace of model development, a standing platform-based benchmarking capability, such as was used in this study, would let a service re-evaluate models and prompts as they evolve.

## Conclusion

Across 23 contemporary LLMs, the accuracy of rheumatology referral urgency triage depended strongly on both model capability and prompt design. Under a simple prompt, larger and more expensive models performed best and accuracy tracked cost. An advanced prompt substantially improved smaller and older model performance, reduced between-model variance 5.3-fold and eliminated the accuracy-cost relationship, indicating that advanced prompting can partially substitute for the inherent reasoning advantage of larger models. Set against published benchmarks, the leading models performed within the range reported for human referral triage, which is itself imperfect and only moderately reproducible, and their agreement with our expert panel approached the level of agreement between the rheumatologists themselves. Triage is a substantial administrative burden that consumes rheumatologist time without advancing patient care. These data suggest that automating it to approximately a human standard may be technically feasible at modest cost, but they are a benchmarking study on constructed cases: prospective evaluation on consecutive real referrals, with defined governance and human oversight of under-triage, is required before clinical use.

## Data Availability

The 20 referral vignettes, the five-category triage definitions, both prompt texts, the independent rheumatologist ratings and the complete per-attempt model outputs (2760 attempts, including category, rationale, latency, token counts and serving cost) are available in a public repository (https://doi.org/10.5281/zenodo.21754374). All model evaluations were performed in July 2026 via the OpenRouter application programming interface aggregator.

## Appendix 1. Referral vignettes and expert consensus categories

The 20 constructed referral vignettes are reproduced below with the consensus urgency category assigned by the four blinded rheumatologists. Where two categories are shown, the expert panel concluded that either represented safe triage and a model was credited for selecting either.

### Case 1

consensus: Semi-Urgent. 50yo male with episodes of gout affecting both feet over the last few years. Attacks last a few days to a week and normal in between. Having a few attacks each year. I started allopurinol a few months ago but this made the gout attacks much worse. Allopurinol was stopped and switched to febuxostat which also worsened the gout attacks. He’s taking Naproxen 1 g SR daily, colchicine 2 daily and having intermittent short courses of prednisolone to resolve attacks. He works as a self-employed landscape gardener has to miss work when he has attacks. Otherwise he has controlled diabetes and hypertension. His medications are colchicine 1 mg daily, Naproxen 1 g SR daily prn, Prednisolone 50 mg daily prn. Metformin 1 g bd, Perindopril 10 mg daily. His FBC, UEC, LFT, CRP are normal. Hi serum urate is 0.65mmol/L.

### Case 2

consensus: Emergency. 60yo female presents with a few months of fatigue, a few weeks of drenching night sweats and 5 kg unintentional weight loss. In the last few days she has developed painful red papules on her legs. She has been having an occasional dry cough without SOBOE. She has not noticed fevers. CXR showed patchy infiltrates. RF and CCP negative. ANA 640, ANCA positive with Pr3 > 100. CRP 55 mg/L.Hb 103 g/L, WCC normal, platelet count 561 × 10⁹/L. Urinalysis was normal.

### Case 3

consensus: Urgent or Semi-Urgent. 38-year-old female with a 15 year history of psoriasis presents with joint pain and swelling over the past year, predominantly in her knees, PIPs and DIPs of the hands. She is still able to do her regular swimming which she enjoys as a sport. She is managing her job and family commitments with only minimal disruption from the pains. She also had chronic fungal-like changes in her nails. The left knee was mildly swollen. No spinal pain. HLA-B27 negative. X-rays of knees showed an effusion with normal joint space. X-rays of the hands show mild joint space of a few PIPJs without erosions. RF 22, CCP negative, ANA 160, FBC normal, ALP mildly elevated otherwise ELFT normal, CRP 20 mg/L. Her pains have partially responded to Meloxicam 15 mg daily. She didn’t tolerate prednisolone 25 mg daily. Could this be rheumatoid arthritis?

### Case 4

consensus: Semi-Urgent or Routine. 27-year-old male with low back pain and stiffness for 3 months. The stiffness is worse in the morning and warms up over a few hours. There is also pain with activity. There is no family history of ankylosing spondylitis. FBC and ELFTs are normal. CRP was 11. HLA-B27 was positive. MRI of the lumbar spine shows moderate left sided sacroiliitis without erosions. There were no other lumbar spine abnormalities. I started long acting naproxen with a PPI and his symptoms are well controlled. He is having regular physiotherapy.

### Case 5

consensus: Routine. 55-year-old female has dry eyes and mouth for the past year or 2. Though her mouth is dry, she is able to swallow food without difficulty finding that sips of water help. She regularly uses tear replacement drops. She has seen an ophthalmologist and had a positive Schirmer’s test. She also has mild generalised joint pain and doesn’t report joint swelling. On examination she had swollen parotid glands and was tender in some of her joints without joint swelling. She is using intermittent NSAID for the joint pains. FBC, ELFT, CMP, CRP were normal. ANA was positive at 1:160 with a positive anti-Ro/SSA and anti-La/SSB. Urinalysis was negative.

### Case 6

consensus: Routine. 68-year-old female patient with progressive right knee pain and swelling over the past 3 months. The pain is localized to the medial aspect of the knee and is exacerbated by weight-bearing activities and prolonged standing. She reports occasional locking sensations and instability, which have caused her to stumble on a few occasions. The patient’s medical history is significant for hypertension, managed with lisinopril, and obesity (BMI 32). She denies any recent trauma to the knee. On examination, there is a moderate effusion and tenderness along the medial joint line. She was able to flex to 110 degrees with full extension. Neurovascular status of the limb is intact. Plain radiographs of the right knee demonstrate moderate medial compartment joint space narrowing and osteophyte formation. There is also evidence of patellofemoral osteoarthritis. MRI concluded moderate degenerate changes, a moderate effusion, and a complex degenerative tear of the medial meniscus.

### Case 7

consensus: Emergency or Hyper-Urgent. A 30-year-old male with a 4-month history of generalized fatigue, malar rash, and intermittent arthralgia, oral ulcers and photosensitivity. His ANA titer was 1:1280 with a speckled pattern. dsDNA was 28. C4 low, C3 mildly reduced. There’s a mild leukopenia of 3.5 × 10^9/L with reduced lymphocytes and normal neutrophils. Itherwise FBC, UEC, and LFT were normal. Urinalysis shows 3 + protein and 3 + RBC.

### Case 8

consensus: Routine. A 40-year-old female with arthralgia and myalgias for a few years. The pains affect her sleep. She also has persistent marked fatigue. She had widespread tenderness but otherwise her examination was normal. FBC, UEC, LFT, CRP, TSH, Vit D were all normal. ANA was 1:320. RF and CCP were negative. Urinalysis was clear. Her past history includes depression which is controlled, and irritable bowel syndrome which is ongoing. She has been seeing a physiotherapist but is not able to exercise as this worsens her pains. She reports allergies (nausea and headache) to paracetamol, NSAIDs, tramal, endep, Lyrica, and gabapentin. She is taking regular oxycodone which she finds helps a little. She also takes escitalopram. The pains have meant she has been unable to work. She is applying for a disability pension and the government agency is requesting a specialist report.

### Case 9

consensus: Routine. A 65-year-old female presents with bilateral knee pain worsening over the past 2 years, more pronounced on the right. She has a BMI of 32 and reports some 20 min of morning stiffness but pains mostly worse with walking, stairs and getting out of the car. Examination showed tenderness around the knee and it didn’t look swollen. X-rays show mild joint space narrowing with early osteophytes in both knees, worse in the right. FBC, UEC, LFT, CRP, B27, ANA, RF, CCP are all negative. She is taking regular paracetamol and mobic 15 mg daily but it isn’t really working. She’d like advice on further management.

### Case 10

consensus: Urgent or Semi-Urgent. 24yo female with generalised joint pain, stiffness, and swelling for 5 months, worse first thing in the morning. Affecting her hands, shoulders, knees, feet. Difficulty with her ADLs and has been taking some time off her work as a process worker because of the pains. Mobic is partially helping. I’ve started 10 mg daily of prednisolone which has helped a lot. She’s otherwise well. Examination showed some soft tissue swelling of her wrists and MCPJs. FBC, ELFT, TSH, uric acid, ANA normal. RF was 35 and CCP negative. X-rays of the hands and feet was normal.

### Case 11

consensus: Urgent or Semi-Urgent. 19yo female with 2 years of raynauds, now 3 weeks rash over her trunk and limbs. No facial rash. Otherwise feels well. Examination showed active raynaud’s and macular rash over trunk and limbs. FBC, ELFT normal. CRP 1. ANA 640, ENA Sm + Ro+ La + RNP+, dsDNA negative. ANCA negative. ACE negative. RF negative. CCP negative. Skin biopsy showed a leucocytoclastic vasculitis with staining for C3 and IgG. I started a weaning course of prednisolone and the rash has resolved. Urinalysis was normal.

### Case 12

consensus: Urgent or Semi-Urgent. 33 male with recurrent severe attacks of acute anterior uveitis of right eye without visual loss. HLAB27+. Serum ACE elevated. RF, ANA, ANCA all negative. CXR and lumbar spine X-rays normal. He denies any other systemic symptoms or back pain. Could you assess him for an underlying rheumatological condition.

### Case 13

consensus: Emergency. 67 male with a few weeks of generalised aches and gradually worsening headaches. Partial response to ibuprofen. FBC and UEC normal. CRP 52. ESR 73. One episode of blurry vision in the right eye which resolved over a few minutes. I’ve started 50 mg of prednisolone.

### Case 14

consensus: Urgent or Semi-Urgent. 71 female with a few months of muscle aches around the shoulders and hips. She also feels fatigued and has lost 3 kg in weight though she is still obese with BMI of 34. Paracetamol didn’t help. She had a partial improvement with meloxicam 15 mg daily. I’ve started 10 mg daily of prednisolone which has largely resolved her symptoms. Her past history: type 2 DM, OA both knees with TKRs right in 2021 and left in 2003, hypertension, CKD stage 3. Her vaccines and cancer screening is up to date. Medications: gliclazide, metformin, perindopril, aspirin, hydrochlorothiazide, meloxicam. FBC normal. Creatinine 122 stable. LFT normal. CRP 43. ESR 66.

### Case 15

consensus: Emergency. 26 yo male with a painful right knee for a few days. He wondered if he’d sprained it at footy on Saturday. Woke with pain and swelling in the knee on Sunday and pain has been increasing since then. He’s having difficulty walking on the knee, using crutches. He feels feverish and has lost his appetite. No rigors. Examination shows a very swollen red knee which is very painful to move through a very limited range. The knee feels hot. PHx: nil. I’ve sent him for blood tests but don’t have the results yet. I’ve started colchicine and naproxen. His uncle has gout so I thought this would be gout.

### Case 16

consensus: Urgent. 45yo female with 3–4 months of pain and stiffness in both hands and wrists, worse in the mornings and struggling with typing and opening jars. She thought it might be menopause or overuse as she also gets intermittent tingling in the fingers at night. On exam there was some fullness at the MCPJs and wrists but no obvious deformity. FBC shows mild anaemia, CRP 28, RF positive, CCP strongly positive, ANA negative. Hand X-rays didn’t show much yet. Meloxicam has helped a bit but she is still having difficulty working.

### Case 17

consensus: Semi-Urgent. 52yo female with longstanding cold painful fingers, now saying the skin on the hands feels tight over recent months. She mainly came to see me because of reflux and poor sleep, and mentioned occasional food “sticking” which she thought was just indigestion. No real joint swelling. Examination showed bp 120/76, shiny tight skin over the fingers and a small sore area on one fingertip. ANA positive, creatinine normal, other bloods largely unremarkable. I started nifedipine with partial benefit for the fingers.

### Case 18

consensus: Emergency. 72yo male with acute painful swollen right knee for 2 days, no clear trauma though he had been doing some gardening. Similar episode in the wrist last year which settled and was put down to a sprain. He has a family history of gout and assumed this was the same, but serum urate is normal. Exam shows a hot effused knee with reduced movement. CRP 242, FBC otherwise unremarkable. Knee X-ray mild chondrocalcinosis and mild OA. Prednisolone has helped somewhat as he can’t take much NSAID with his renal impairment.

### Case 19

consensus: Semi-Urgent. 29yo male with swollen right knee and sore left ankle for about 3 weeks. He first thought he’d overdone it at the gym, though he also a red eye that settled with some drops from the chemist. No psoriasis that I could see. He is otherwise fit and well. CRP 38, RF/CCP negative, HLA-B27 pending. Naproxen has taken the edge off but he is still limping.

### Case 20

consensus: Hyper-Urgent or Urgent. 26yo female with 6 weeks of fevers, fatigue, sore throat and intermittent rash. She has been in and out of our local regional hospital and treated with antibiotics twice which haven’t helped. Joint pains mainly affect wrists, knees and ankles, but not constantly. She looked generally unwell on examination and had some mild generalised lymphadenopathy. FBC shows neutrophilia, CRP 113. ANA and RF negative, LFT mild derangement. The regional hospital suggested a rheumatology opinion.
